# A two-layered machine learning method to identify protein O-GlcNAcylation sites with O-GlcNAc transferase substrate motifs

**DOI:** 10.1186/1471-2105-16-S18-S10

**Published:** 2015-12-09

**Authors:** Hui-Ju Kao, Chien-Hsun Huang, Neil Arvin Bretaña, Cheng-Tsung Lu, Kai-Yao Huang, Shun-Long Weng, Tzong-Yi Lee

**Affiliations:** 1 Department of Computer Science and Engineering, Yuan Ze University, Taoyuan 320, Taiwan; 2 Tao-Yuan Hospital, Ministry of Health & Welfare, Taoyuan 320, Taiwan; 3 Inflammation and Infection Research Centre, School of Medical Sciences, University of New South Wales, Sydney, Australia; 4 Department of Obstetrics and Gynecology, Hsinchu Mackay Memorial Hospital, Hsin-Chu 300, Taiwan; 5 Mackay Junior College of Medicine, Nursing and Management, Taipei 112, Taiwan; 6 Department of Medicine, Mackay Medical College, New Taipei City 252, Taiwan; 7 Innovation Center for Big Data and Digital Convergence, Yuan Ze University, Taoyuan 320, Taiwan

**Keywords:** O-GlcNAcylation, O-linked glycosylation, O-GlcNAc transferase (OGT), substrate motif, profile hidden Markov model, support vector machine

## Abstract

Protein O-GlcNAcylation, involving the β-attachment of single *N*-acetylglucosamine (GlcNAc) to the hydroxyl group of serine or threonine residues, is an O-linked glycosylation catalyzed by O-GlcNAc transferase (OGT). Molecular level investigation of the basis for OGT's substrate specificity should aid understanding how O-GlcNAc contributes to diverse cellular processes. Due to an increasing number of O-GlcNAcylated peptides with site-specific information identified by mass spectrometry (MS)-based proteomics, we were motivated to characterize substrate site motifs of O-GlcNAc transferases. In this investigation, a non-redundant dataset of 410 experimentally verified O-GlcNAcylation sites were manually extracted from dbOGAP, OGlycBase and UniProtKB. After detection of conserved motifs by using maximal dependence decomposition, profile hidden Markov model (profile HMM) was adopted to learn a first-layered model for each identified OGT substrate motif. Support Vector Machine (SVM) was then used to generate a second-layered model learned from the output values of profile HMMs in first layer. The two-layered predictive model was evaluated using a five-fold cross validation which yielded a sensitivity of 85.4%, a specificity of 84.1%, and an accuracy of 84.7%. Additionally, an independent testing set from PhosphoSitePlus, which was really non-homologous to the training data of predictive model, was used to demonstrate that the proposed method could provide a promising accuracy (84.05%) and outperform other O-GlcNAcylation site prediction tools. A case study indicated that the proposed method could be a feasible means of conducting preliminary analyses of protein O-GlcNAcylation and has been implemented as a web-based system, OGTSite, which is now freely available at http://csb.cse.yzu.edu.tw/OGTSite/.

## Introduction

A type of O-linked glycosylation, Protein O-GlcNAcylation (O-GlcNAc), attaches a single N-acetylglucosamine (GlcNAc) to serine (Ser)/threonine (Thr) residues [1]. O-GlcNAc, commonly found on cytoplasmic and nuclear proteins, has been shown to modulate molecular processes and cellular processes [2]. O-GlcNAc transferase (OGT) is an enzyme responsible for the addition of O-GlcNAc during glycosylation. On the other hand, an enzyme O-GlcNAcase (OGA) can remove O-GlcNAc. Recently, extracellular O-linked β-N-acetylglucosamine (EOGT) [3], an atypical OGT, has been reported to be responsible for extracellular O-GlcNAcylation of secreted and membrane glycoproteins [4]. Protein O-GlcNAcylation is also responsible for regulating cell-cell and cell-matrix interactions [5]. Accumulating evidence suggests that OGTs may act as a nutrient sensor that links hexosamine biosynthesis pathway to oncogenic signaling and regulation of factors involved in glucose and lipid metabolism [6]. The O-GlcNAc-dependent regulation seems to play an important role in the signaling pathways involved in metabolic reprograming of cancer cells [7]. In addition, O-GlcNAcylation is also an important post-translational modification and deregulation of this mechanism has been linked to various diseases such as diabetes [8], Alzheimer disease [9] and cancers [10-12].

With the improvement in mass spectrometry technologies, O-GlcNAcylated proteins in postsynaptic density [13], murine synapse [14], mouse brain [15], rat brain [16], mouse embryonic stem cell [17], and Hela cells [18], have been identified in recent years. However, precise identification of O-GlcNAcylation sites remains to be a challenge due to its dynamic characteristics [19]. Due to an interest to better identify O-GlcNAcylation sites and reduce experimental efforts, computational prediction of site motifs and O-GlcNAcylation sites have been considered. Previously, Gupta and Brunak have developed YinOYang - an O-GlcNAcylation prediction tool trained using 40 O-GlcNAcylation sites [20]. Chen et al. have developed a similar tool incorporating structural topology to identify O-glycosylation sites on transmembrane proteins [21]. The increase in experimentally identified O-GlcNAcylation sites motivates new developments including OGlcNAcScan, which was trained using 373 O-GlcNAcylation sites [22]. More recently, a new prediction tool, O-GlcNAcPRED, has been proposed claiming to have better performance than the aforementioned tools [23]. In the midst of these developments, Carage et al. have demonstrated that ensembles of support vector machine (SVM) classifiers could outperform single SVM classifier in terms of predicting protein glycosylation sites [24].

Although several computational methods have been developed to predict protein O-GlcNAcylation sites, there is currently no such tool that includes the investigation of potential OGT substrate motifs. It has been reported that molecular level investigation on OGT substrate specificity may aid in understanding how O-GlcNAc contributes to a diverse set of cellular processes [25]. With this, we were motivated to characterize O-GlcNAcylation sites with the consideration of amino acid composition [26]. In this study, we apply maximal dependence decomposition (MDD) to explore potential OGT substrate motifs for the experimentally verified O-GlcNAcylation sites. Statistically significant substrate motifs were further tested its prediction power by cross-validation evaluation and independent testing. A two-layered machine learning method, incorporating profile hidden Markov model (HMM) and support vector machine (SVM), was utilized to construct the predictive models. Furthermore, to facilitate the study of protein O-GlcNAcylation, MDD-identified substrate motifs were exploited to implement a web-based tool for identifying O-GlcNAcylation sites with corresponding OGT substrate motifs.

## Material and methods

### Construction of positive and negative training data sets

Due to the high-throughput mass spectrometry-based glycol-proteomics [27], several databases [22,28-30] have been developed for cumulating experimentally verified O-GlcNAcylation sites by manually surveying the glycosylation-associated literatures. In this work, the data set for training the predictive model of O-GlcNAcylation sites was mainly extracted from dbOGAP [22], O-GlycBase [31], and UniProtKB [32]. From dbOGAP, a total of 250 and 142 sites for O-GlcNAcylated serine (Ser) and threonine (Thr) on 172 proteins were collected. From O-GlycBase version 6.0, 24 sites for O-GlcNAcylated Ser and Thr from 17 proteins were collected. In UniProtKB, experimentally verified O-GlcNAcylation data were first filtered by removing entries annotated as "by similarity", "potential", "probable". This resulted to the collection of 66 and 51 sites for O-GlcNAcylated Ser and Thr on 53 proteins. To avoid data redundancy, each data obtained from one database was compared to the data obtained from the other databases based on its O-GlcNAcylated site position and the UniProtKB accession number utilized by all three databases. Redundancy was removed by retaining only one record in the event of finding multiple records of the same site position and accession number. After the removal of redundant data, we have obtained 261 and 149 non-redundant sites for O-GlcNAcylated Ser and Thr on 176 proteins.

As shown in Table 1 the combined non-redundant data of 410 experimentally verified O-GlcNAcylation sites from dbOGAP, OGlycBase and UniProtKB was regarded as the positive data for the investigation of OGT substrate motifs and the construction of predictive models. With an attempt to explore the substrate motifs of O-GlcNAc transferases, sequence fragments were extracted using a window length of 11 centered on O-GlcNAcylated Ser and Thr residues [33,34]. In this investigation, the sequence fragments centered on non-O-GlcNAcylated Ser and Thr residues were regarded as negative training data. After removing identical sequence fragments, a total of 17381 and 10587 negative sequence fragments for Ser and Thr residues were obtained from 176 O-GlcNAcylated proteins.

**Table 1:** Data statistics of positive and negative training data.

Data resource	Residue	Number of O-GlcNAcylated sites (Positive data)	Number of non-O-GlcNAcylated sites (Negative data)	Number of non-O-GlcNAcylated sites (Balanced negative data)
dbOGAP	Serine	250	18,570	-
	
	Threonine	142	11,240	-

OGlycBase	Serine	24	1,013	-
	
	Threonine	24	694	-

UniProtKB	Serine	66	4,851	-
	
	Threonine	51	3,255	-

**Non-redundant dataset**	Serine	261	17,381	261
	
	Threonine	149	10,587	149
	
	Combined	410	27,968	410

### Detection of OGT substrate motifs

**Figure 1:**
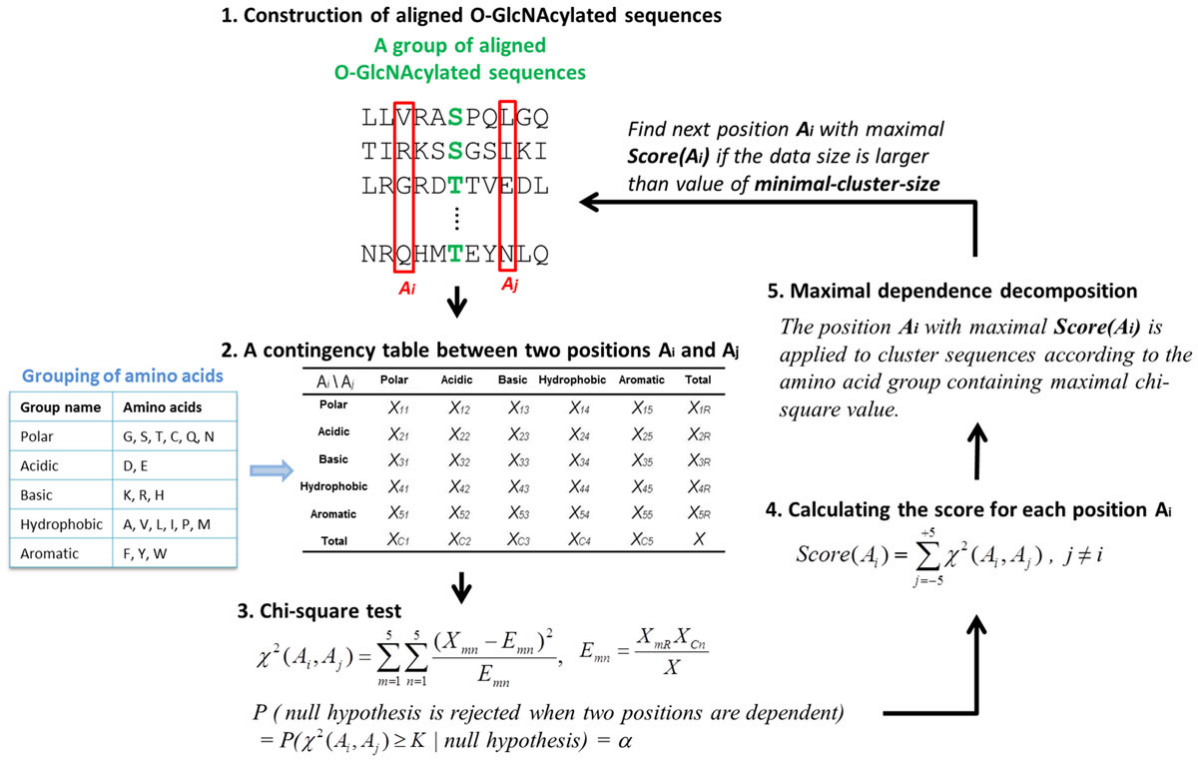
**Analytical flowchart of MDD clustering**.

The O-GlcNAc transferase (OGT) exhibits substrate site specificity for the sugar donor recognition mechanism and the interaction to target proteins [35]. In this investigation, a recursively statistical method, maximal dependence decomposition (MDD) [36], was applied to the positive training data in order to discover substrate motif signatures of O-GlcNAcylation sites by clustering a large-scale dataset of aligned sequences into subgroups that contain statistically significant substrate motifs. MDD extract motifs according to the conserved biochemical property of amino acids. In order to do this, the twenty types of amino acids are categorized into five groups: polar, acidic, basic, hydrophobic, and aromatic groups, as shown in Table S1 (Additional file 1). A contingency table of the amino acids occurrence between two positions is then constructed, as presented in Figure 1. MDD utilizes chi-squared test to test the dependence of amino acid occurrence between two positions, *A_i _*and *A_j_*, surrounding the O-GlcNAcylated site [37]. The chi-squared test implemented in MDD is defined as:

(1) $$\chi^{2}\left({\text{A}}_{\text{i}},{\text{A}}_{\text{j}}\right)=\overset{5}{\underset{m=1}{\sum }}\overset{5}{\underset{n=1}{\sum }}\frac{{\left(X_{mn}-E_{mn}\right)}^{2}}{E_{mn}}$$

where *X_mn _*represented the number of sequences having amino acids from group *m *in position *A_i _*and amino acids from group *n *in position *A_j_*, for each pair (*A_i _, A_j_*) with *i*≠j. *E_mn _*is calculated as $\frac{X_{mR}\cdot X_{Cn}}{X}$, where *X_mR _*= *X_m1_*+ ...+*X_m5_, X_Cn _*= *X_1n_*+ ...+*X_5n_*, and *X *denotes the total number of sequences. If a strong dependence is detected (defined as that the chi-square value was larger than 34.3, corresponding to a cutoff level of *P *= 0.005 with 16 degrees of freedom) between two positions, then the process is continued as described [38]. Moreover, a minimum cluster size is set when applying MDD to cluster the sequences in the positive training data. If the data size of a subgroup was less than the given parameter, the subgroup will not be divided any further. For this study, MDD was executed using various values in order to obtain an optimal minimum cluster size.

### Construction of two-layered prediction model

**Figure 2:**
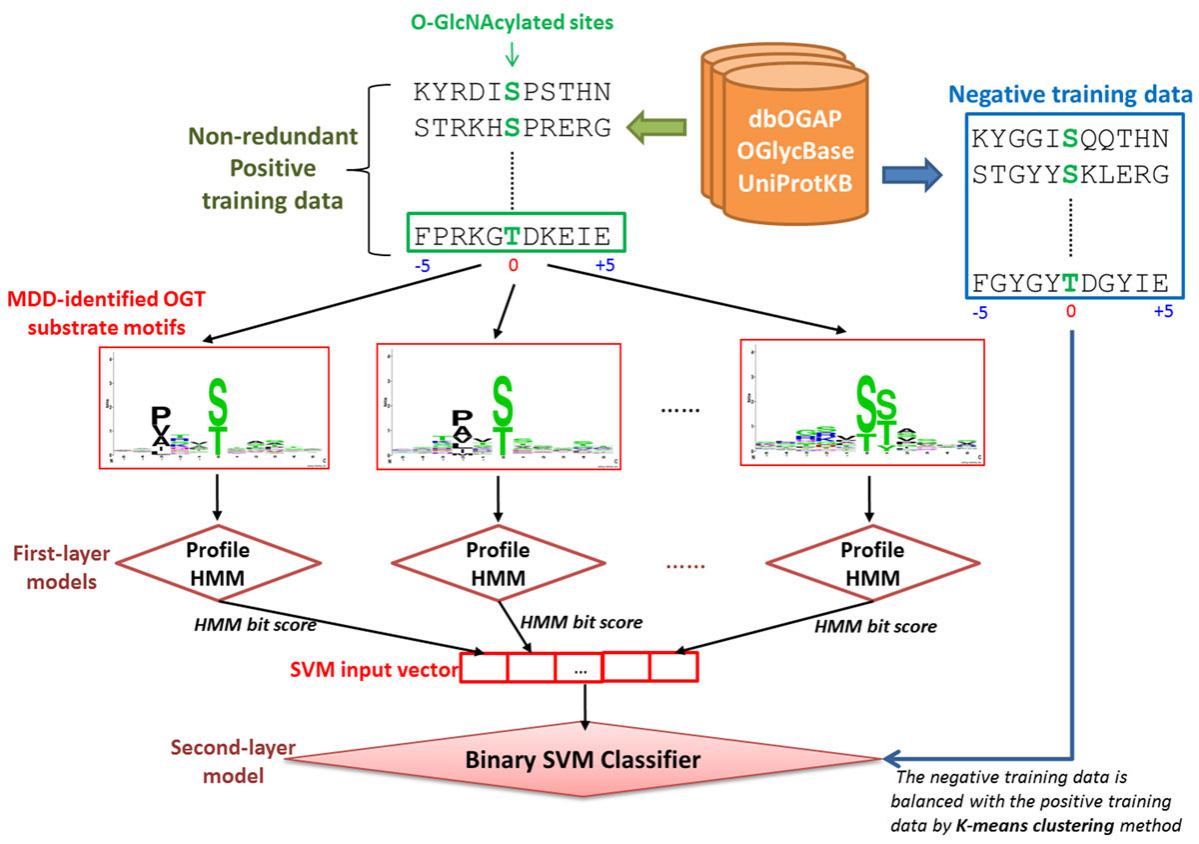
**Conceptual diagram of constructing two-layered prediction model from MDD-identified substrate motifs**.

In this work, the two-layered machine learning method, incorporating profile hidden Markov model (HMM) and support vector machine (SVM), was used to construct the predictive model from the positive data and negative data of the training set. As presented in Figure 2, profile HMM is generated for each MDD-clustered subgroup in first layer. After applying MDD clustering on O-GlcNAcylated data, the sequence fragments of each MDD-clustered subgroup is taken as a training set to build a profile HMM. An HMM detects distant relationships between amino acid sequences by describing a probability distribution over a potentially infinite number of sequences [39]. In this study, we utilized the software package HMMER [39] in order to build profile HMMs, to calibrate the HMMs, and to search putative O-GlcNAcylation sites against the protein sequences. As models are built based on positive instances of a class, only positive data are utilized to build a predictive model. For each model of the MDD-clustered subgroups, a threshold parameter is selected for identifying potential positive sites from a query [39]. The optimal threshold is the value that gives the most optimal cross-validation performance for each training model. For every search, HMMER returns a bit score and an expectation value (E-value) for each sequence fragment. The bit score is the base two logarithm of the ratio between the probability that the query sequence is a significant match and the probability that the query is produced by a random model. Additionally, the E-value represents the expected number of sequences with a score greater than or equal to the returned HMMER bit scores. A search result with an HMMER bit score greater than the threshold parameter is taken as a positive prediction. While decreasing the bit score threshold favors finding true positives, increasing the bit score threshold favors finding true negatives. Therefore, the threshold must be optimized to obtain a balanced number of true positives and true negatives.

In second layer, a binary SVM classifier is trained using the bit scores of profile HMMs. Based on binary classification, SVMs map the input samples into a higher dimensional space using a kernel function. It then finds a hyper-plane that discriminates between the two classes with maximal margin and minimal error. For this study, we employed a public SVM library, LIBSVM [40], to generate the second-layered model from the bit scores of positive and negative training data. The radial basis function (RBF) $K\left(S_{i},S_{j}\right)=\text{exp}\left(-\gamma ||S_{i}-S_{j}|{|}^{2}\right)$ was used as the kernel function of the SVM. The LIBSVM library is able to produce a probability ranging from 0 to 1 for each prediction; in default, a probability value higher than 0.5 is defined as a positive instance. In order to avoid a biased prediction performance, the negative training data was balanced with the positive training data. To select a representative set of negative data, *K*-means clustering [36,41] was employed with reference to previous PTM prediction methods [42-47]. This resulted in an equal number of positive and negative sequence fragments for the training data (Table 1).

### Five-fold cross validation and performance evaluation

Five-fold cross validation was performed in order to evaluate the predictive performance of each model using various parameters. For this process, the training data is divided into five groups by splitting each dataset into approximately equal sized subgroups where one subgroup is regarded as the test set while the remaining four subgroups are regarded as the training set. This process is repeated five times with each subgroup being used as a test set once [48]. The following measures were used to gauge the average predictive performance of the trained models: Sensitivity (Sn) = TP / (TP+FN), Specificity (Sp) = TN / (TN+FP), Accuracy (Acc) = (TP + TN) / (TP+FP+TN+FN), and Matthews Correlation Coefficient $\left(\text{MCC}\right)=\frac{\left(TP\times TN\right)-\left(FN\times FP\right)}{\sqrt{\left(TP+FN\right)\times \left(TN+FP\right)\times \left(TP+FP\right)\times \left(TN+FN\right)}}$, where TP, TN, FP and FN represent the numbers of true positives, true negatives, false positives and false negatives, respectively. After thirty rounds of cross-validation process, average Sn, Sp, Acc and MCC values were calculated for each model. The predictive model with the best average performance was then selected for further evaluation by independent testing dataset.

### Construction of independent testing data set

In order to address a potential overestimation of the predictive performance of the models due to over-fitting, an independent test was carried out. For this analysis, experimentally validated sequences obtained from PhosphoSitePlus [49] were used as independent testing data. A total of 779 and 582 experimentally verified sites for O-GlcNAcylated Ser and Thr on 542 proteins were obtained from PhosphoSitePlus. Similar to the construction of positive training set, the sequence fragments centered on O-GlcNAcylated Ser and Thr residues are extracted using 11-mer window length. Additionally, O-GlcNAcylated sequence fragments homologous to the positive training data were removed in order to generate a non-homologous independent testing data. As a result, a total of 956 sequence fragments, consisting of 522 and 434 O-GlcNAcylated Ser and Thr residues, respectively, were regarded as the positive data for independent testing. On the other hand, sequence fragments centered on non-O-GlcNAcylated Ser and Thr residues were regarded as negative data for independent testing. Upon removing homologous data, a total of 60976 sequence fragments (38682 and 22294 non-O-GlcNAcylated Ser and Thr residues) were collected for the negative testing data.

## Results and discussion

### Amino acids composition of O-GlcNAcylation sites

**Figure 3:**
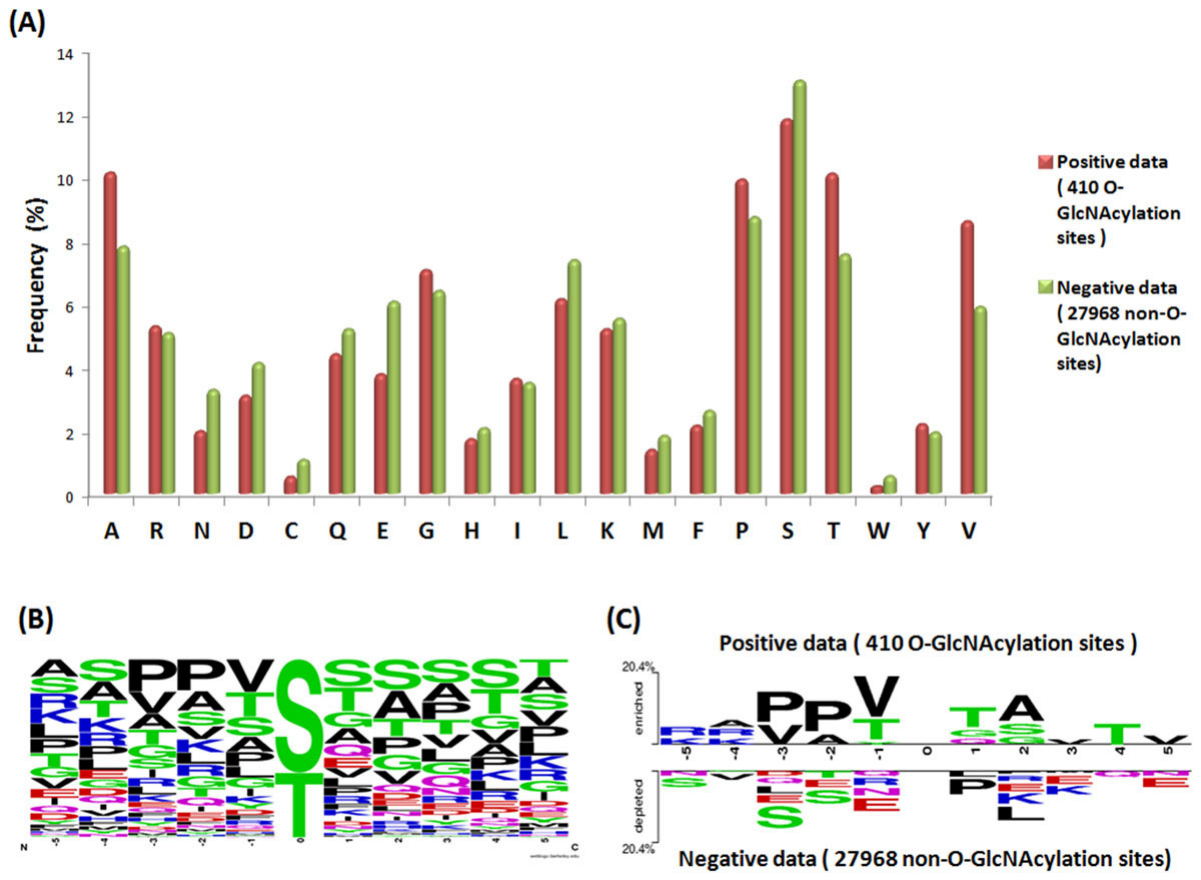
**Amino acids composition surrounding the O-GlcNAcylation sites**. (A) Comparison of amino acids composition between positive data (410 O-GlcNAcylation sites) and negative data (27968 non-O-GlcNAcylation sites). (B) Position-specific amino acids composition surrounding the O-GlcNAcylation sites. (C) TwoSampleLogo (*p*-value<0.05) between positive data and negative data.

This study aims to investigate the OGT substrate motifs based on the amino acid composition surrounding O-GlcNAcylation sites. Figure 3(A) presents the comparison of amino acids composition between positive data (410 O-GlcNAcylated sites) and negative data (27968 non-O-GlcNAcylated sites). O-GlcNAcylated sites appear to conatin more hydrophobic amino acids than non-O-GlcNAcylated sites. On the other hand, non-O-GlcNAcylated sites appear to contain more charged amino acids than O-GlcNAcylated sites. Polar amino acids appear to be well distributed in both data sets. The position-specific amino acids composition surrounding the O-GlcNAcylation sites is visualized using WebLogo as shown in Figure 3(B). O-GlcNAcylated Ser/Thr (positive data) residues and unmodified ones (negative data) were centered on position 0, and the flanking amino acids (-5~+5). The difference between the amino acid composition of O-GlcNAcylated and non-O-GlcNAcylated sites is further visualized using TwoSampleLogo [50], as shown Figure 3(C). It can be clearly observed that the most pronounced feature of O-GlcNAcylation sites is the abundance of hydrophobic amino acids Proline (P), Valine (V), and Alanine (A), locating centrally around position -2 and +3. Besides, the polar amino acids, Threonine (T) and Serine (S), also located centrally at position -1 and +1. Additionally, charged amino acids, especially the positively charged Lysine (K) and Arginine (R) were dominant at position -2, -4 and -5, suggesting that the distant amino acids in sequence, which may be close to O-GlcNAcylation sites in three-dimensional structure, showed notable difference between modified and unmodified sites. Another featured characteristic is the depletion of P and L at +1 and +2, respectively, which is immediately adjacent to the O-GlcNAcylation sites. It should also be noted that S, T, and Glutamate (E) were also found to be less frequent around position -2, -3, and +5.

### Substrate site motifs of O-GlcNAc transferases

**Figure 4:**
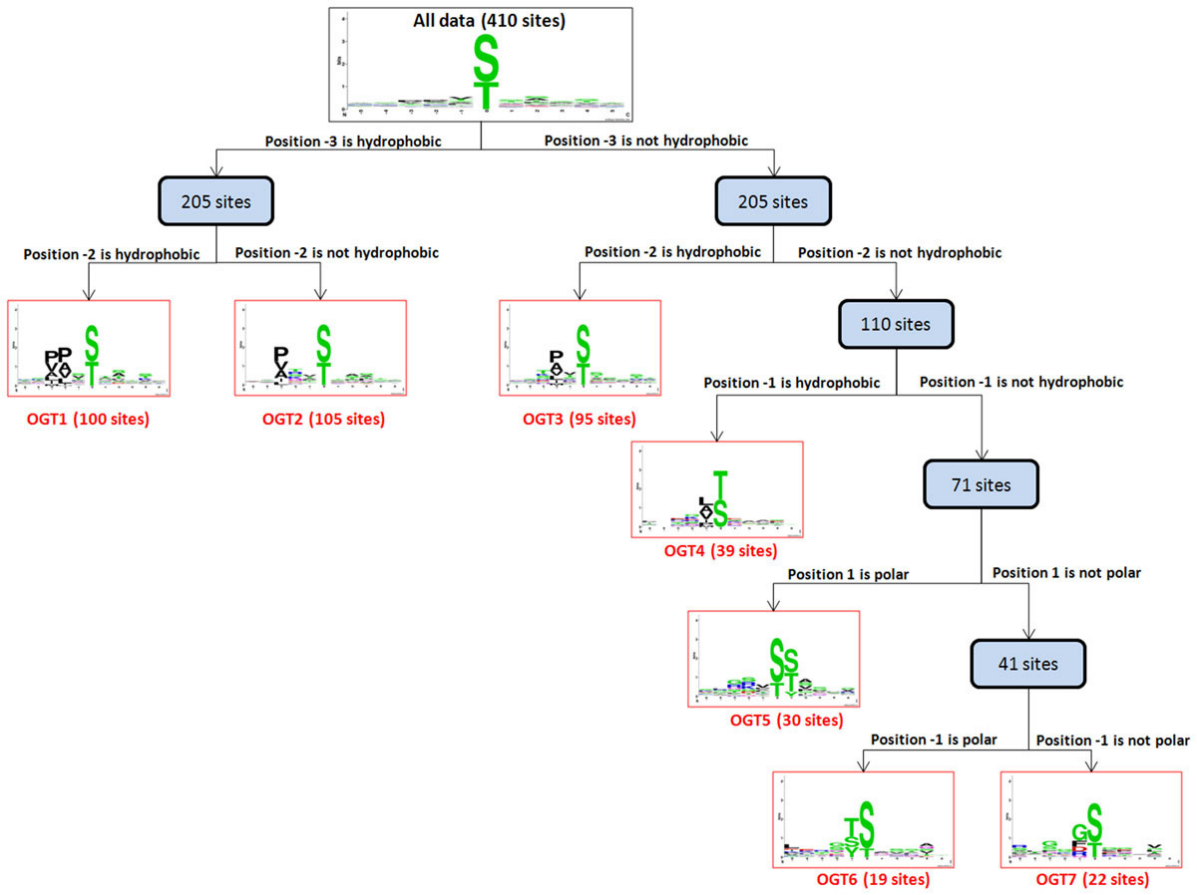
**The tree view of potential OGT substrate motifs identified by MDD clustering on 410 O-GlcNAcylation sites**.

This study focuses on the characterization of potential OGT substrate motifs based on the amino acid composition surrounding O-GlcNAcylation sites. In order to detect the potential OGT substrate motifs from large scale O-GlcNAcylation data set, we applied the MDD to further cluster all 410 experimentally verified O-GlcNAcylated peptide sequences into subgroups by iteratively capturing the positions with maximal dependence of amino acids composition. As illustrated in Figure 4, the MDD-identified substrate motifs were visualized in a tree-like structure. Firstly, position -3 had the maximal dependence with the occurrence of hydrophobic amino acid group. Subsequently, all 205 sites containing the hydrophobic group in position -3 could be further divided into two subgroups: subgroup OGT1 (100 sites) represented the occurrence of hydrophobic amino acids in position -2 with maximal dependence, whereas subgroup OGT2 (105 sites) had no occurrence of hydrophobic amino acids in position -2. It would be noticed that the subgroup OGT1 gives the substrate motif of hydrophobic amino acids in both positions -3 and -2, which is consistent with the consensus motif previously suggested as P-P-T-[ST]-T-A [22]. In right subtree, the data (205 sites) without the hydrophobic amino acids in position -3 were divided into two subgroups: subgroup OGT3 (95 sites) involved the maximal dependence of hydrophobic amino acids in position -2, yet the other (110 sites) had no occurrence of hydrophobic amino acids in position -2. Furthermore, the 110 sites containing could be divided into two subgroups: subgroup OGT4 (39 sites) represented the occurrence of hydrophobic amino acids in position -1 while the other (71 sites) had no occurrence of hydrophobic amino acids in position -1. The hydrophobic residues indicate its contribution in the interfaces of protein-protein interactions. Finally, totally seven OGT substrate motifs (marked in red) were identified with significant dependences (*P*-value < 0.005). Subgroups OGT5 and OGT6 depicted the conserved motifs of polar amino acids at positions +1 and -1, respectively. However, subgroup OGT7, that contains the remaining 22 O-GlcNAcylation sites, had a little conserved motif of glycine (G) residue at position -1. Interestingly, the small size and flexibility of G residue is probably responsible for making it suitable for the structural adjustments required during the protein-protein interactions [51]. Table S2 (Additional file 2) shows the number of O-GlcNAcylation sites (positive data) in each MDD-identified OGT substrate motif.

### Predictive performance of the identified substrate motifs

To identify how to best classify O-GlcNAcylation from non-O-GlcNAcylation sites, the predictive models were trained with each of the following: OGT1, OGT2, OGT3, OGT4, OGT5, OGT6, OGT7, as well as all OGTs combined. The predictive power of each model was evaluated by measuring the sensitivity (Sn), specificity (Sp), accuracy (Acc), and Matthews correlation coefficient (MCC). According to evaluation of five-fold cross-validation, the single HMM trained from all 410 positive data yield a sensitivity of 68.8%, a specificity of 70.7%, an accuracy of 69.8%, and an MCC value of 0.395. As shown in Table 2 the HMM trained from OGT1 subgroup, that contains a conserved motif of hydrophobic amino acids at positions -2 and -3, provided the highest predictive power with a sensitivity of 93.0%, a specificity of 89.0%, an accuracy of 91.0%, and an MCC value of 0.821. Among all others, the HMM trained from OGT4 subgroup yielded the lowest sensitivity 0.71.8%, while the HMM trained from OGT7 subgroup yielded the lowest specificity at 68.2%. Among the seven subgroups, the HMM trained from OGT7 subgroup provided a lowest accuracy at 70.5%. Additionally, combining seven OGT HMMs (MDD-clustered HMMs) could achieve the predictive performance of 83.7% sensitivity, 77.1% specificity, 80.4% accuracy, and 0.609 MCC value. This investigation indicated that the application of MDD clustering could improve the performance on the prediction of protein O-GlcNAcylation sites.

**Table 2:** Five-fold cross validation results on profile HMMs learned from all data and seven MDD-clustered subgroups.

Models	Number of positive data	Number of negative data	Sn	Sp	Acc	MCC
Single HMM with all data	410	410	68.8%	70.7%	69.8%	0.395

HMM with OGT1	100	100	93.0%	89.0%	91.0%	0.821

HMM with OGT2	105	105	83.8%	71.4%	77.6%	0.557

HMM with OGT3	95	95	85.3%	75.8%	80.5%	0.613

HMM with OGT4	39	39	71.8%	74.4%	73.1%	0.462

HMM with OGT5	30	30	73.3%	73.3%	73.3%	0.467

HMM with OGT6	19	19	78.9%	73.7%	76.3%	0.527

HMM with OGT7	22	22	72.7%	68.2%	70.5%	0.410

MDD-clustered HMMs (Combined 7 OGT HMMs)	410	410	83.7%	77.1%	80.4%	0.609

Two-layered model (7 HMMs + 1 SVM)	410	410	85.4%	84.1%	84.7%	0.695

With reference to a previous work applying two-layered SVMs on the prediction of viral phosphorylation sites [52], this work further combined seven profile HMMs (first layer) and one SVM (second layer) into a two-layered prediction model, which provides a better performance than the combination of seven OGT HMMs (MDD-clustered HMMs). The two-layered prediction model yielded a sensitivity of 85.4%, a specificity of 84.1%, an accuracy of 0.84.7%, and an MCC value of 0.695. In this investigation, the model providing best performance was further evaluated by independent testing set.

### Independent testing and comparison with other prediction tools

The final non-redundant data of independent testing set consisting of 956 positive sites and 60976 negative sites was utilized for further evaluating the constructed models. As shown in Table 3 the single HMM trained using all positive data achieved a sensitivity of 63.70%, a specificity of 65.72%, an accuracy of 65.69%, and an MCC value of 0.076. The MDD-clustered HMMs achieved a sensitivity of 87.13%, a specificity of 74.15%, an accuracy of 74.38%, and an MCC value of 0.171. This investigation indicated that a greater prediction power could be obtained by using MDD-clustered HMM models than that by a single HMM model. Additionally, the two-layered model achieved a sensitivity of 86.61%, a specificity of 84.01%, an accuracy of 84.05%, and an MCC value of 0.231. The independent testing demonstrated that the two-layered model could perform better than MDD-clustered HMMs and could provide a promising accuracy for 542 experimentally verified O-GlcNAcylated proteins, which were not considered within the construction of predictive model.

**Table 3:** The comparison of independent testing results between our methods and other three O-GlcNAcylation prediction tools.

Methods	TP	FN	TN	FP	Sn	Sp	Acc	MCC
Single HMM with all data	609	347	40072	20904	63.70%	65.72%	65.69%	0.076

MDD-clustered HMMs (7 OGT HMMs)	833	123	45212	15764	87.13%	74.15%	74.38%	0.171

Two-layered model (7 HMMs + 1 SVM)	828	128	51224	9752	86.61%	84.01%	84.05%	0.231

YinOYang	449	507	50619	10357	46.97%	83.01%	82.46%	0.097

O-GlcNAcScan	411	545	51219	9757	42.99%	84.00%	83.37%	0.089

O-GlcNAcPRED	554	402	38414	22562	57.95%	63.00%	62.92%	0.053

To further demonstrate the effectiveness of our method, the independent testing set was used to compare the two-layered model with three popular O-GlcNAcylation site prediction tools, YinOYang, O-GlcNAcScan, and O-GlcNAcPRED. Table 3 indicated that the prediction power yielded by our two-layered model was superior to that by other three prediction tools. By using default threshold value (0.5), YinOYang yielded a sensitivity of 46.97%, a specificity of 83.01%, an accuracy of 82.46%, and an MCC value of 0.097. O-GlcNAcScan achieved a sensitivity of 42.99%, a specificity of 84.00%, an accuracy of 83.37%, and an MCC value of 0.089. O-GlcNAcPRED provided a lowest independent testing performance: 57.95% sensitivity, 63.00% specificity, 62.92% accuracy, and 0.053 MCC value. This independent testing indicated that the two-layered model could provide balanced sensitivity and specificity for such unbalanced positive and negative datasets. The proposed method also provided comparable accuracy with that analyzed by O-GlcNAcScan. Overall, as presented in Figure S1 (Additional file 3), the proposed method outperformed the three prediction tools.

### Web-based system for the identification of O-GlcNAcylation sites

**Figure 5:**
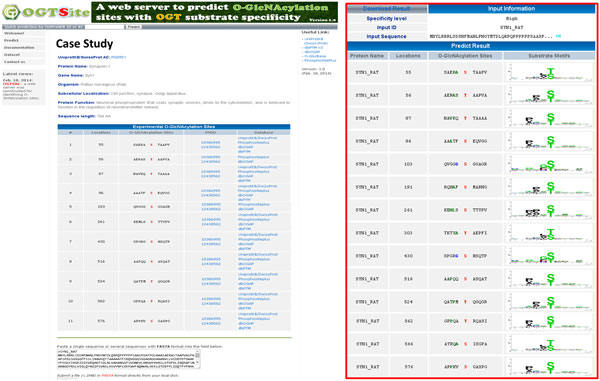
**A case study of O-GlcNAcylation sites prediction on Synapsin-1 (Syn1) of Rattus norvegicus**.

With the time-consuming and lab-intensive experimental identification of protein O-GlcNAcylation sites, a biologist may only concluded that a protein can be O-GlcNAcylated but the precise O-GlcNAcylation sites remains unknown. Therefore, an effective prediction server can help to focus efficiently on potential sites. After evaluation by cross-validation and independent testing, the two-layered model with best predictive performance was adopted to implement a web-based system, named OGTSite, for predicting O-GlcNAcylated sites with potential OGT substrate motifs. As shown in Figure 5, users can submit their protein sequences in FASTA format or just provide the UniProtKB accession number. The system returns the predictions, including O-GlcNAcylated position and flanking amino acids. Users can also access the substrate motifs used for predicting the O-GlcNAcylation sites. Take Synapsin-1 (Syn1) of Rattus norvegicus as an example, 11 sites such as S55, T56, T87, S96, S103, S261, S430, S516, T524, T562 and S576 have been experimentally verified as O-GlcNAcylation sites [53]. OGTSite predicted a total of 14 potential O-GlcNAcylation sites, including 11 true positive predictions. Although S191, T303 and T566 have not yet been validated as the O-GlcNAcylation sites, they have the potential OGT3 and OGT4 substrate motifs, respectively. This case study suggests the feasibility of this model to identify the S/T residues that can be modified by O-GlcNAc moiety.

## Conclusion

This study presents a novel scheme to identify potential substrate specificity of O-GlcNAc transferase based on a set of experimentally verified O-GlcNAcylation sites. We have demonstrated the utility of MDD clustering method in the characterization of substrate motifs of O-GlcNAcylation sites. Additionally, the proposed pipeline includes the effectiveness of the identified MDD-detected short linear motifs to predict O-GlcNAcylated sites. A five-fold cross-validation evaluation showed the power of MDD-identified substrate motifs in the prediction of O-GlcNAcylated sites. Moreover, the two-layered model combining seven profile HMMs and one SVM could provide the best performance. The two-layered model has been used to implement an online system, OGTSite, for an effective identification of protein O-GlcNAcylation sites. By identifying potential O-GlcNAcylation sites using the proposed method, we will be providing a reliable lead to the scientific community to minimize costs and effort for experimentally verifying actual O-GlcNAcylation sites. It should be noted that the proposed method could also be extended to include more meaningful substrate motifs by further acquiring experimentally verified O-GlcNAcylation sites. Additionally, a more abundant set of experimentally verified O-GlcNAcylation sites with protein tertiary structure information could be used to strengthen site prediction capabilities [54].

## Competing interests

The authors declare that they have no competing interests.

## Authors' contributions

TYL and SLW conceived and supervised the project. HJK, CHH, CTL and KYH were responsible for the design, computational analyses, implemented the web-based tool, and drafted the manuscript with revisions provided by NAB and TYL. All authors read and approved the final manuscript.

## Availability

The proposed method is implemented as a web-based resource, which is now freely available to all interested users at http://csb.cse.yzu.edu.tw/OGTSite/. All of the dataset used in this work is also available for download in the website.

## Supplementary Material

Additional file 1 **Table S1**. The grouping of twenty amino acids used in this study. Click here for file

Additional file 2 **Table S2**. The identified OGT substrate motifs of 410 O-GlcNAcylation sites Click here for file

Additional file 3 **Figure S1**. The comparison of independent testing results between our methods and other three O-GlcNAcylation prediction tools. Click here for file

## Declarations

Publication charge for this work was funded by MOST grant under contract number of MOST 103-2221-E-155-020-MY3 and MOST 104-2221-E-155-036-MY2 to TYL.

This article has been published as part of *BMC Bioinformatics *Volume 16 Supplement 18, 2015: Joint 26th Genome Informatics Workshop and 14th International Conference on Bioinformatics: Bioinformatics. The full contents of the supplement are available online at http://www.biomedcentral.com/bmcbioinformatics/supplements/16/S18.
