# Supplementary material for: A two-layered machine learning method to identify protein O-GlcNAcylation sites with O-GlcNAc transferase substrate motifs
Source: BMC Bioinformatics. 2015 Dec 9;16(Suppl 18):S10. doi: 10.1186/1471-2105-16-S18-S10 (PMC4682369; doi:10.1186/1471-2105-16-S18-S10)
Supplement: Additional file 1 — Table S1. The grouping of twenty amino acids used in this study. [file 1471-2105-16-S18-S10-S1.pdf]

Table S1. The grouping of twenty amino acids used in this study.

Chemical properties	Amino acids
**Polar group**	Glycine (G), Serine (S), Threonine (T), Cysteine (C), Glutamine (Q), Asparagine (N)
**Acidic group**	Aspartic acid (D), Glutamic acid (E)
**Basic group**	Lysine (K), Arginine (R), Histidine (H)
**Hydrophobic group**	Alanine (A), Valine (V), Leucine (L), Isoleucine (I), Proline (P), Methionine (M)
**Aromatic group**	Phenylalanine (F), Tyrosine (Y), Tryptophan (W)
