# Supplementary material for: A two-layered machine learning method to identify protein O-GlcNAcylation sites with O-GlcNAc transferase substrate motifs
Source: BMC Bioinformatics. 2015 Dec 9;16(Suppl 18):S10. doi: 10.1186/1471-2105-16-S18-S10 (PMC4682369; doi:10.1186/1471-2105-16-S18-S10)
Supplement: Additional file 2 — Table S2. The identified OGT substrate motifs of 410 O-GlcNAcylation sites [file 1471-2105-16-S18-S10-S2.pdf]

**Table S2. The identified OGT substrate motifs of 410 O-GlcNAcylation sites.**

| Substrate groups | Entropy plot of sequence logo | Number of O-GlcNAcylation sites |
|---|---|---|
| All data | 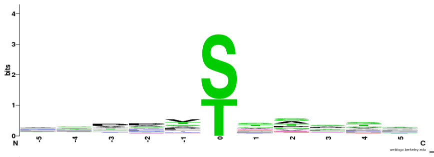 | 410 |
| OGT1 | 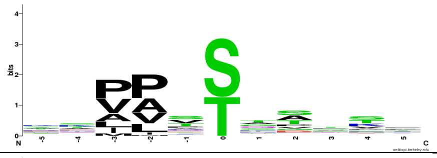 | 100 |
| OGT2 | 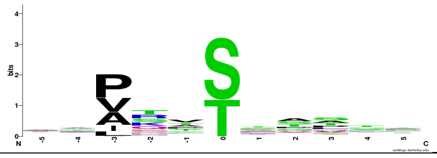 | 105 |
| OGT3 | 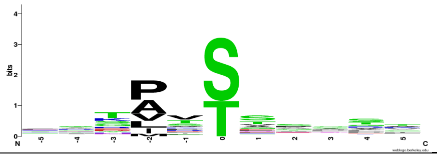 | 95 |
| OGT4 | 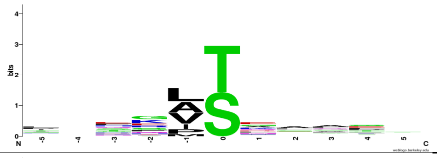 | 39 |
| OGT5 | 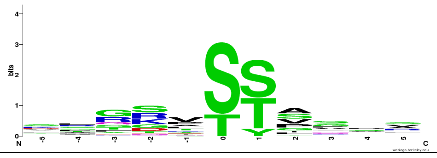 | 30 |
| OGT6 | 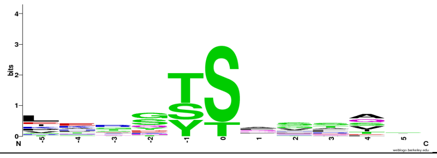 | 19 |
| OGT7 | 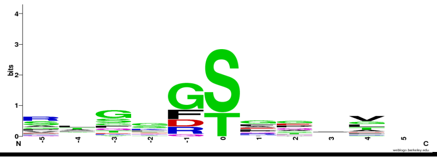 | 22 |
