# Supplementary material for: A two-layered machine learning method to identify protein O-GlcNAcylation sites with O-GlcNAc transferase substrate motifs
Source: BMC Bioinformatics. 2015 Dec 9;16(Suppl 18):S10. doi: 10.1186/1471-2105-16-S18-S10 (PMC4682369; doi:10.1186/1471-2105-16-S18-S10)
Supplement: Additional file 3 — Figure S1. The comparison of independent testing results between our methods and other three O-GlcNAcylation prediction tools. [file 1471-2105-16-S18-S10-S3.pdf]

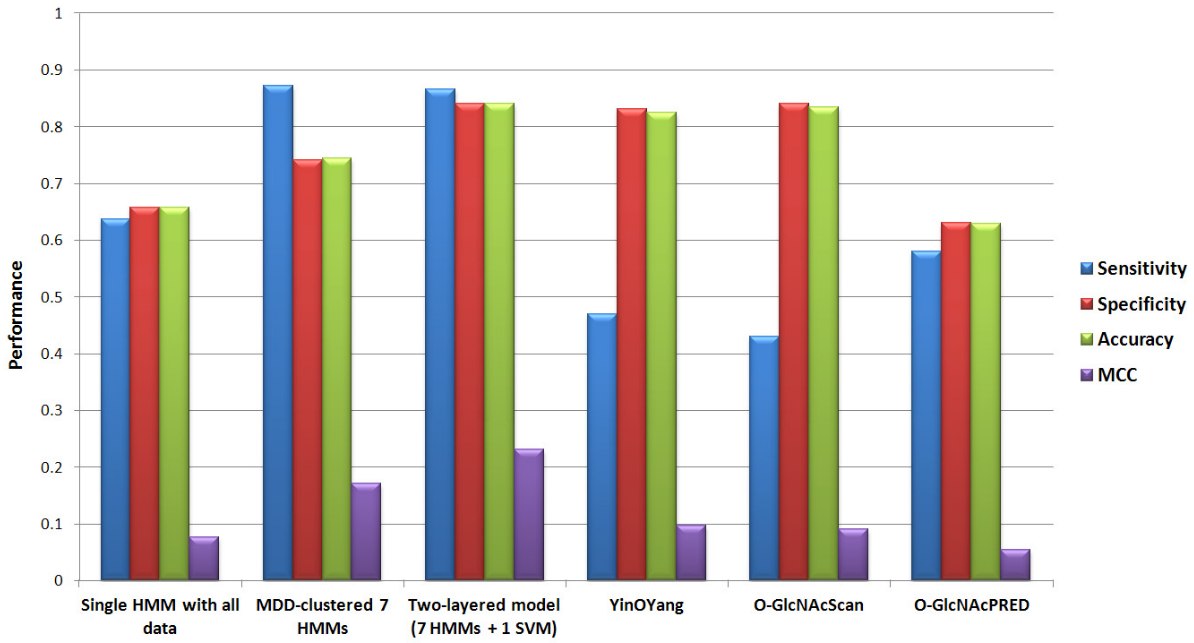

Figure S1. The comparison of independent testing results between our methods and other three O-GlcNAcylation prediction tools.
